# Supplementary material for: The gut microbiome in konzo
Source: Nat Commun. 2021 Sep 10;12:5371. doi: 10.1038/s41467-021-25694-1 (PMC8433213; doi:10.1038/s41467-021-25694-1)
Supplement: Supplementary file 1 — Supplementary Information [file 41467_2021_25694_MOESM1_ESM.pdf]

Supplementary Information

The Gut Microbiome in Konzo

Matthew S. Bramble[1§], Neerja Vashist[1,2§], Arthur Ko[3], Sambhawa Priya[4], Céleste Musasa[1], Alban Mathieu[5], D'Andre Spencer[1], Michel Lupamba-Kasendue [6], Patrick Mamona-Dilufwasayo [1,6], Kevin Karume[1,6], Joanna Nsibu[6], Hans Manya[1,6], Mary N.A. Uy[7], Brian Colwell[8], Michael Boivin[9], J.P Banae Mayambu[10], Daniel Okitundu[11], Arnaud Droit[5], Dieudonné Mumba-Ngoyi[6,12], Ran Blekhman[4], Desire Tshala-Katumbay[6,13]*, Eric Vilain[1,2,14]*

Affiliations:
[1] Center for Genetic Medicine Research, Children's Research Institute, Children's National Hospital, Washington, D.C., USA.
[2] Department of Genomics and Precision Medicine, George Washington University School of Medicine and Health Sciences, Washington, DC, USA
[3] Department of Medicine, David Geffen School of Medicine, University of California, Los Angeles, Los Angeles, California, USA.
[4] Departments of Genetics, Cell Biology, and Development, University of Minnesota, Minneapolis, MN, USA.
[5] Computational Biology Laboratory, CHU de Québec - Université Laval Research Center, Québec City, QC, Canada
[6] Institut National de Recherche Biomédicale (INRB), Kinshasa, DR. Congo.
[7] College of Medicine, University of the Philippines, Manila, Manila Philippines
[8]School of Public Health, Texas A&M University, College Station, Texas, USA
[9]Department of Psychiatry and Neurology & Ophthalmology, Michigan State University, East Lansing, MI, USA
[10]Ministry of Health National Program on Nutrition (PRONANUT), Kinshasa, DR. Congo
[11]Centre Neuro-Psychopathologique (CNPP), University of Kinshasa, Kinshasa, Congo.
[12] Department of Tropical Medicine, University of Kinshasa, Kinshasa, DR. Congo.
[13]Department of Neurology and School of Public Health, Oregon Health & Science University, Portland, OR, USA.
[14] International Research Laboratory of Epigenetics, Data, Politics, Centre National de la Recherche Scientifique, Washington, DC, USA and Paris, France

§ These authors contributed equally to this work.

* Correspondence should be addressed to Drs. Desire Tshala-Katumbay: tshalad@ohsu.edu and Eric Vilain: evilain@CNMC.org

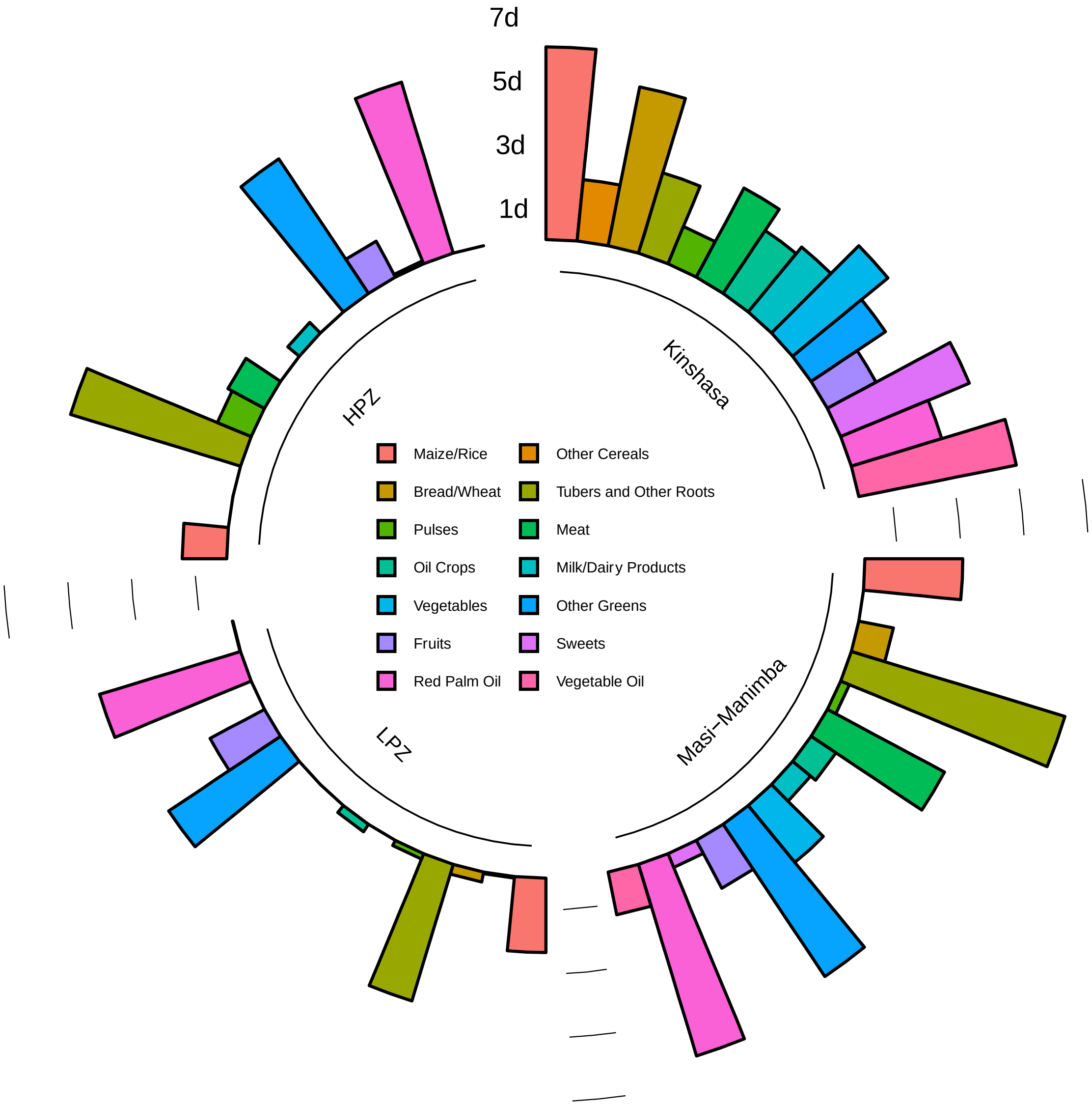

Supplementary Figure 1, Food Diversity Profiles of Study Groups: Average of 7-day recall diet frequency questionnaires from the study participants in Kinshasa, Masi-Manimba and the high prevalence zone (HPZ) and low prevalence zone (LPZ) regions of Kahemba, regardless of disease status or konzo prevalence zone. Corresponding data used to generate supplementary figure 1 can be found in Supplementary Data File 1.

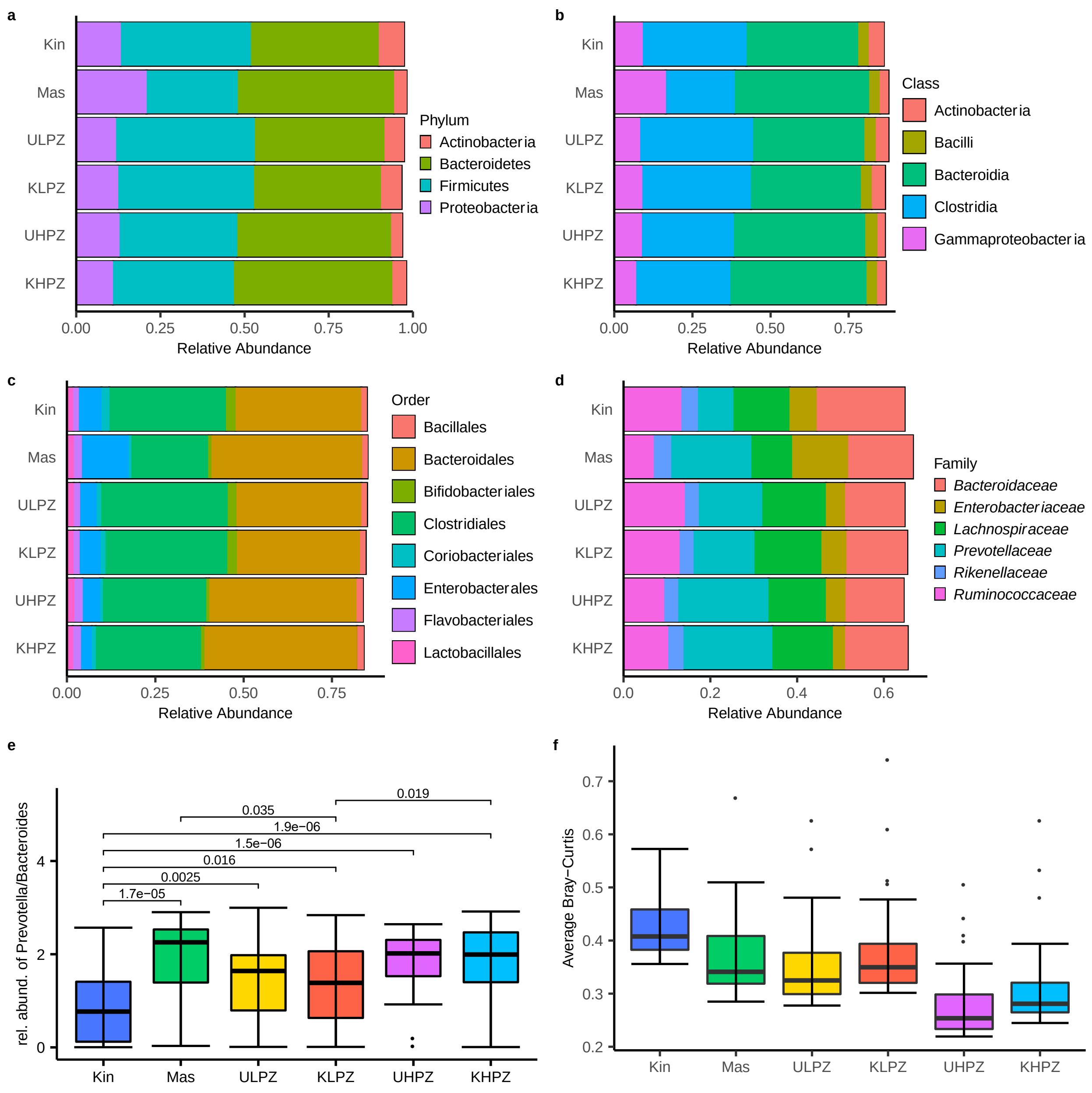

Supplementary Figure 2, Overall Bacterial Distribution Profiles in Study Groups: a-d) Group average of relative abundance for the top most abundant bacterial phylum, class, order and family taxonomic assignments in our study population. Standard deviation and corresponding values for figures A-D can be found in supplementary file 2. **e)** Box plot representation of the ratio of relative abundance for *Prevotella* to *Bacteroides* in each study group (P:B). Statistics are based on pair-wise comparisons of the P:B ratios using the two-sided t-test. **f)** Box plot representation of intra-group Bray-Curtis dissimilarity measures for each study cohort. In e and f, samples are from Kinshasa (Kin) (n=30), Masi-Manimba (Mas) (n=30), Unaffected Low Prevalence Zone (ULPZ) (n=30), Konzo Low Prevalence Zone (KLPZ) (n=30), Unaffected High Prevalence Zone (UHPZ) (n=30), and Konzo High Prevlanece Zone (KHPZ) (n=30). Additionally, data are represented as box plots where the middle line in the box is the median, the lower hinge is the first quartile, the upper hinge is the third quartile, and the whiskers extend from the lower and upper hinges to the smallest and largest value, respectively, at most to 1.5 * IQR (IQR, interquartile range, is the distance between the first and third quartile), and the outliers are plotted individually.

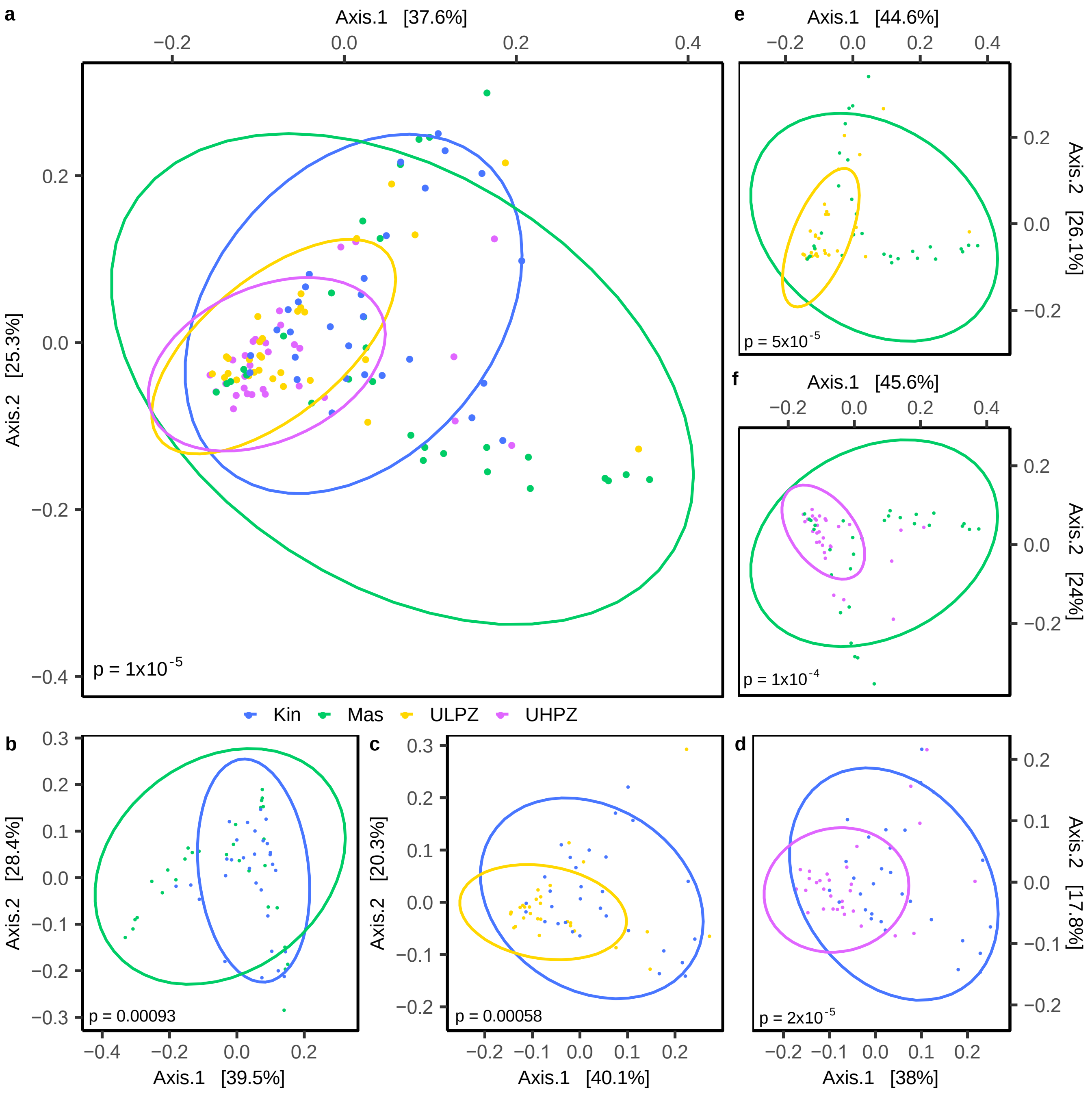

Supplementary Figure 3, Global Measures of Gut Bacteria Dissimilarity at the Functional Level for a Geographic Context: a) PCoA representations based on Bray-Curtis dissimilarity matrix values using the relative abundance of KEGG Orthology (KO) assignments for **a)** Kinshasa (Kin) vs. Masi-Manimba (Mas) and unaffected children from the low prevalence zone (ULPZ) and high prevalence zone (UHPZ) of Kahemba combined, **b)** Kinshasa vs. Masi-Manimba, **c)** Kinshasa vs. ULPZ, **d)** Kinshasa vs. UHPZ, **e)** Masi-Manimba vs. ULPZ and **f)** Masi-Manimba vs. UHPZ. Statistics of Bray-Curtis dissimilarity were generated using PERMANOVA.

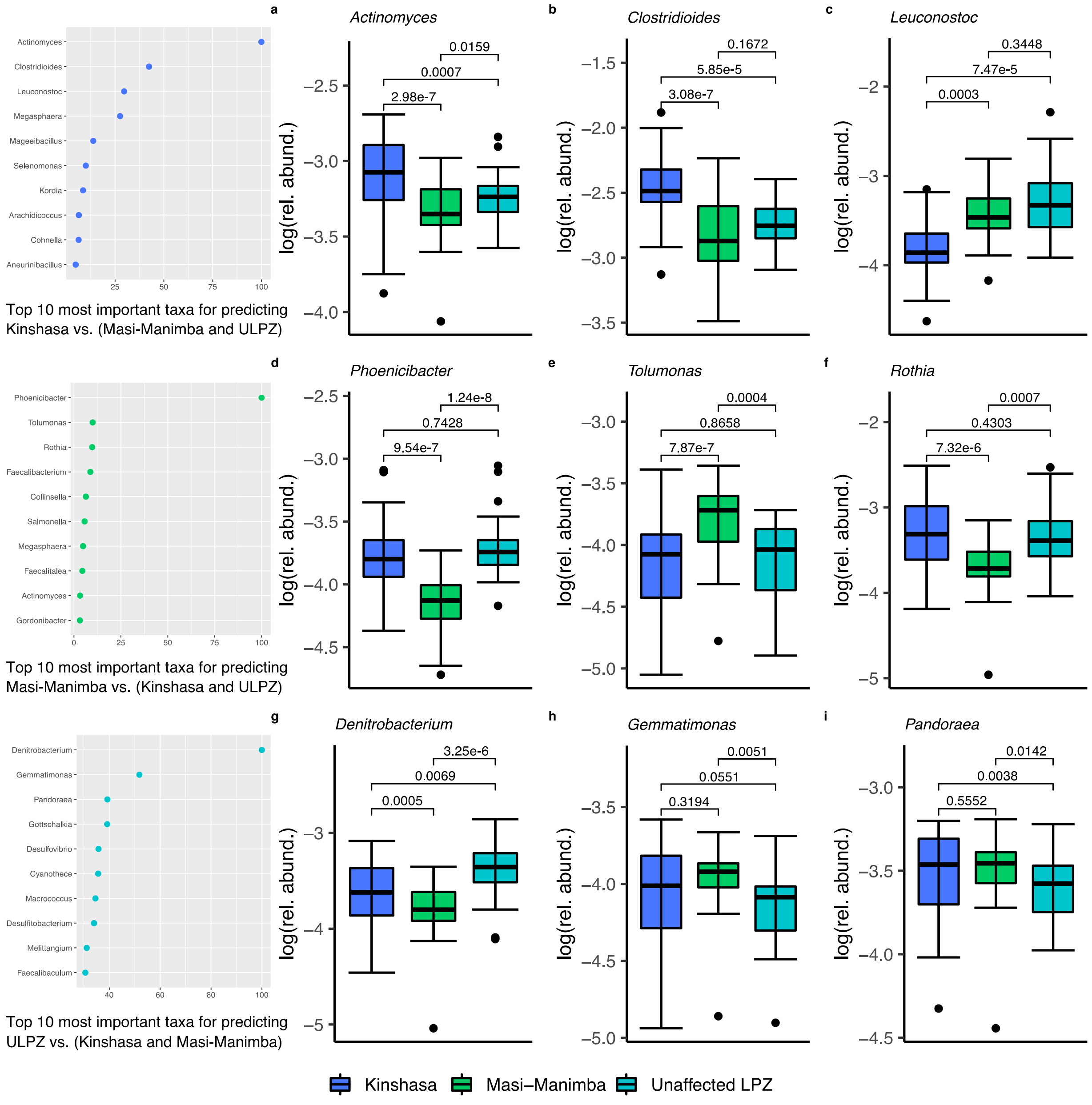

Supplementary Figure 4, Important Taxa for Distinguishing Populations in a Geographic Context: a-i) Box plot representation of the relative abundances for the three most important genera used to distinguish Kinshasa (n = 30) from Masi-Manimba (n = 30) and Unaffected Low Prevalence Zone (ULPZ) (n = 30) (a-c), Masi-Manimba (n = 30) from Kinshasa (n = 30) and ULPZ (n = 30) (d-f) and ULPZ (n = 30) from Masi-Manimba (n = 30) and Kinshasa (n = 30) (g-i) for the Random Forest classifier. Statistics are based on pair-wise comparisons and reported as expected BH corrected p-value FDR<.05, two-sided Wilcoxon test, ALDEx2. In a to i, data are represented as box plots where the middle line in the box is the median, the lower hinge is the first quartile, the upper hinge is the third quartile, and the whiskers extend from the lower and upper hinges to the smallest and largest value, respectively, at most to 1.5 * IQR (IQR, interquartile range, is the distance between the first and third quartile), and the outliers are plotted individually.

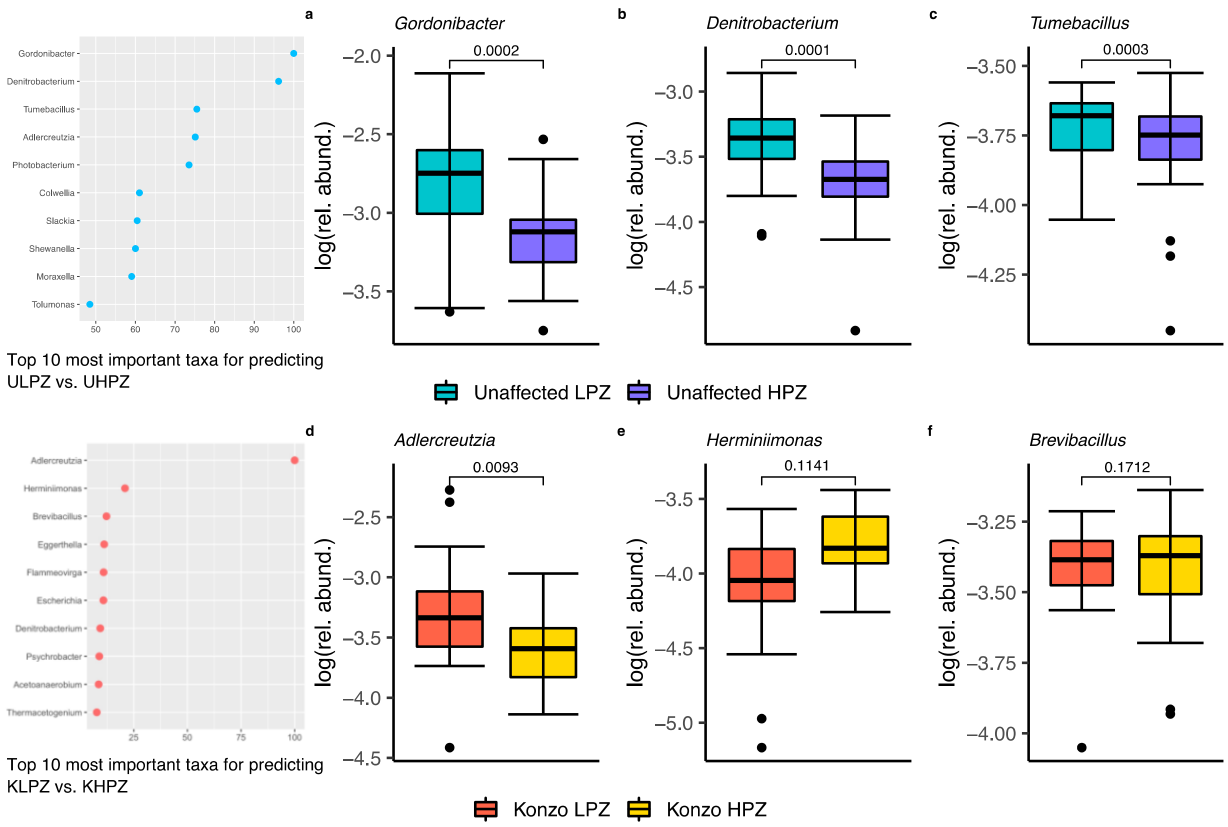

Supplementary Figure 5, Important Taxa for Distinguishing Populations in Kahemba: Box plot representation of the relative abundances for the three most important genera used to distinguish Unaffected Low Prevalence Zone (ULPZ) (n = 30) from Unaffected High Prevalence Zone (UHPZ) (n = 30) (a-c) and Konzo Low Prevalence Zone (KLPZ) (n = 30) from Konzo High Prevalence Zone (KHPZ) (n = 30) (d-f) for the Random Forest classifier. Statistics are based on pair-wise comparisons and reported as expected BH corrected p-value FDR<.05, two-sided Wilcoxon test, ALDEx2. In a to f, data are represented as box plots where the middle line in the box is the median, the lower hinge is the first quartile, the upper hinge is the third quartile, and the whiskers extend from the lower and upper hinges to the smallest and largest value, respectively, at most to 1.5 * IQR (IQR, interquartile range, is the distance between the first and third quartile), and the outliers are plotted individually.

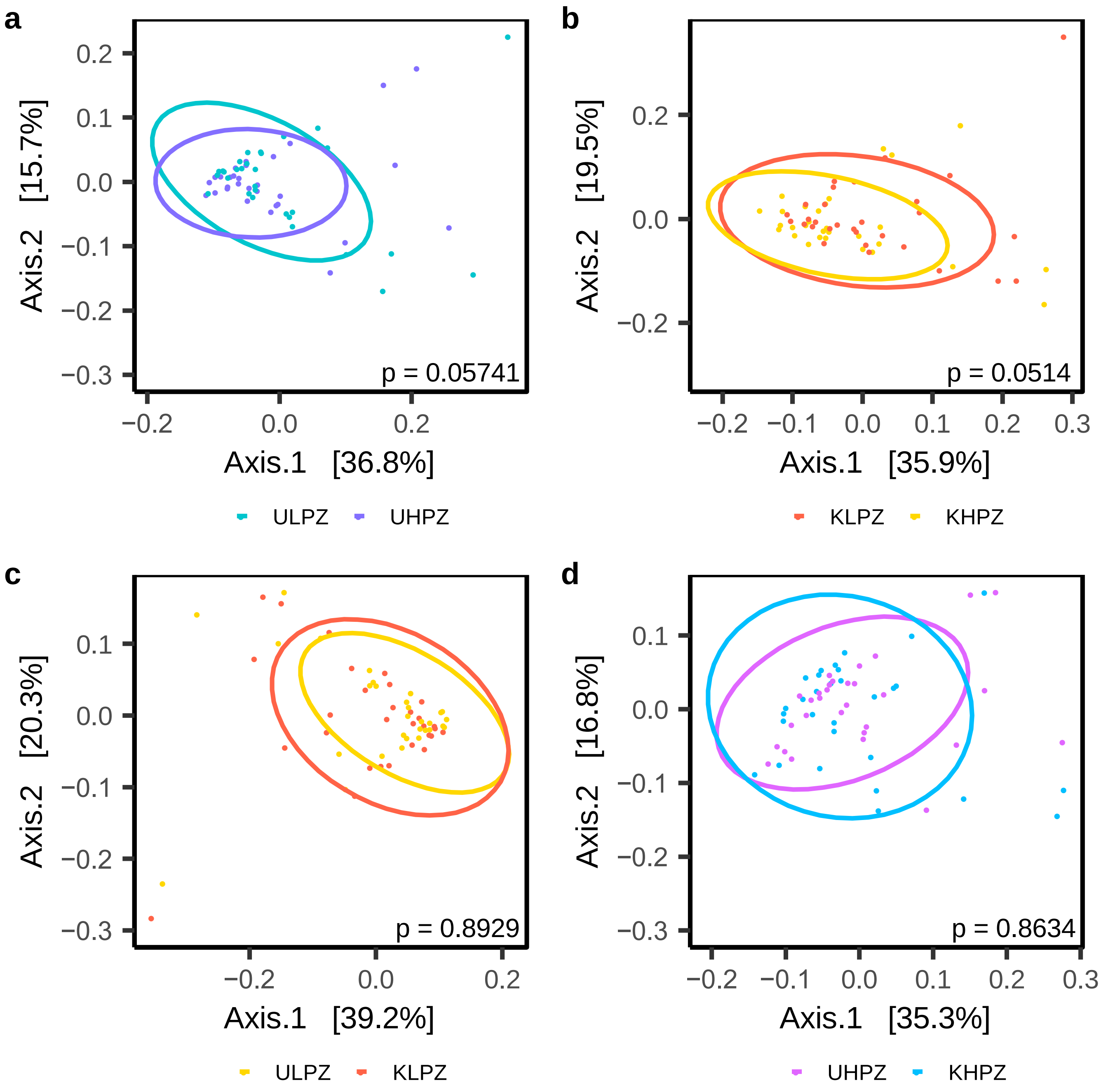

Supplementary Figure 6, Global Measures of Gut Bacteria Dissimilarity at the Functional Level for the Kahemba Region: a) PCoA representations based on Bray-Curtis dissimilarity matrix values using the relative abundance of KEGG Orthology (KO) assignments for **a)** Unaffected children from the low prevalence zone (ULPZ) vs. unaffected children from the high prevalence zone (UHPZ). **b)** Konzo affected children from the low prevalence zone (KLPZ) vs. konzo affected children from the high prevalence zone (KHPZ). **d)** Unaffected children from the LPZ vs. konzo affected children from the LPZ. **d)** Unaffected children from the HPZ vs. konzo affected children from the HPZ. Statistics for Bray-Curtis dissimilarity were generated using PERMANOVA.

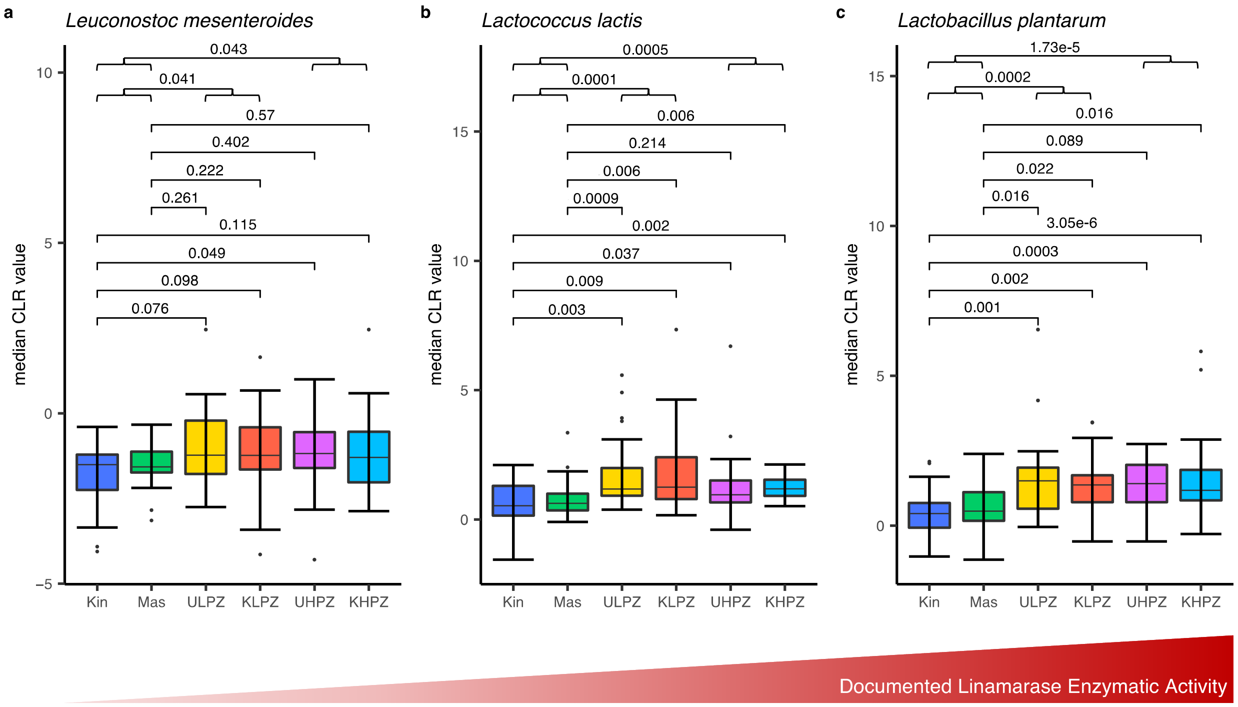

Supplementary Figure 7, Abundance of Linamarase Positive Lactic Acid Bacteria Between Groups of Study: Box plot representations of the median CLR transformed values to compare relative abundance between study groups for **a)** *Leuconostoc mensteroides*, **b)** *Lactococcus lactis* and **c)** *Lactobacillus plantarum*. Statistics are based on pair-wise/group comparisons and reported as expected BH corrected p-value FDR<.05, two-sided Wilcoxon test, using Aldex2 to account for data compositionality. In a to c, samples are from Kinshasa (Kin) (n=30), Masi-Manimba (Mas) (n=30), Unaffected Low Prevalence Zone (ULPZ) (n=30), Konzo Low Prevalence Zone (KLPZ) (n=30), Unaffected High Prevalence Zone (UHPZ) (n=30), and Konzo High Prevalence Zone (KHPZ) (n=30). Additionally, data are represented as box plots where the middle line in the box is the median, the lower hinge is the first quartile, the upper hinge is the third quartile, and the whiskers extend from the lower and upper hinges to the smallest and largest value, respectively, at most to 1.5 * IQR (IQR, interquartile range, is the distance between the first and third quartile), and the outliers are plotted individually.
