# Supplementary material for: The gut microbiome in konzo
Source: Nat Commun. 2021 Sep 10;12:5371. doi: 10.1038/s41467-021-25694-1 (PMC8433213; doi:10.1038/s41467-021-25694-1)
Supplement: Supplementary file 3 — Description of Additional Supplementary Files [file 41467_2021_25694_MOESM3_ESM.docx]

**Title:** Supplementary Data 1.

**Description:** The file contains sample metadata, sample diet data, and the Bacteria read counts for the samples at the various taxonomic ranks.

**Title:** Supplementary Data 2.

**Description:** The file contains associated data with Figure 2 and Supplementary Figure 2, and the Bacteria raw relative abundance values at the various taxonomic ranks (separate sheets with values for bacteria that pass filtration are also included for reference).

**Title:** Supplementary Data 3.

**Description:** The file contains raw and BH corrected p-values after running the Wilcoxon test on the various pairwise comparisons for Bacteria (post filtration) at each taxonomic rank (output after running the aldex function on read counts).

**Title:** Supplementary Data 4.

**Description:** The file contains raw and BH corrected (adjusted) p-values after running the MWW test (Wilcoxon test) on the relative abundance values for various pairwise comparisons for Bacteria (post filtration) at each taxonomic rank.

**Title:** Supplementary Data 5.

**Description:** The file contains the KO read counts for each sample and KO relative abundance values the samples (separate sheet for values of KO that pass filtration for reference).

**Title:** Supplementary Data 6.

**Description:** The file contains raw and BH corrected (adjusted) p-values after running the MWW test (Wilcoxon test) on the relative abundance values for various pairwise comparisons for KOs (post filtration).
