# Supplementary material for: The gut microbiome in konzo
Source: Nat Commun. 2021 Sep 10;12:5371. doi: 10.1038/s41467-021-25694-1 (PMC8433213; doi:10.1038/s41467-021-25694-1)
Supplement: Supplementary file 10 — Reporting Summary [file 41467_2021_25694_MOESM10_ESM.pdf]

nature research

Corresponding author(s): Eric Vilain, M.D., Ph.D. and Desire Tshala Katumbay, M.D., Ph.D.

Last updated by author(s): July 9th, 2021

Reporting Summary

Nature Research wishes to improve the reproducibility of the work that we publish. This form provides structure for consistency and transparency in reporting. For further information on Nature Research policies, see our Editorial Policies and the Editorial Policy Checklist.

Statistics

For all statistical analyses, confirm that the following items are present in the figure legend, table legend, main text, or Methods section.

n/a	Confirmed	
☐	☒	The exact sample size (*n*) for each experimental group/condition, given as a discrete number and unit of measurement
☐	☒	A statement on whether measurements were taken from distinct samples or whether the same sample was measured repeatedly
☐	☒	The statistical test(s) used AND whether they are one- or two-sided<br>*Only common tests should be described solely by name; describe more complex techniques in the Methods section.*
☐	☒	A description of all covariates tested
☐	☒	A description of any assumptions or corrections, such as tests of normality and adjustment for multiple comparisons
☐	☒	A full description of the statistical parameters including central tendency (e.g. means) or other basic estimates (e.g. regression coefficient) AND variation (e.g. standard deviation) or associated estimates of uncertainty (e.g. confidence intervals)
☐	☒	For null hypothesis testing, the test statistic (e.g. *F*, *t*, *r*) with confidence intervals, effect sizes, degrees of freedom and *P* value noted<br>*Give P values as exact values whenever suitable.*
☒	☐	For Bayesian analysis, information on the choice of priors and Markov chain Monte Carlo settings
☒	☐	For hierarchical and complex designs, identification of the appropriate level for tests and full reporting of outcomes
☒	☐	Estimates of effect sizes (e.g. Cohen's *d*, Pearson's *r*), indicating how they were calculated

Our web collection on statistics for biologists contains articles on many of the points above.

Software and code

Policy information about availability of computer code

Data collection	Code utilized in this manuscript does not utilize custom algorithms or software, but the code has been deposited from github and made available on Zenodo (https://doi.org/10.5281/zenodo.5171168) for open access. There was no custom software used in this study but applications that were used to draw conclusions are published and available for public use, and all deviations outside of standard operating procedures were noted in the manuscript.
Data analysis	Code utilized in this manuscript has been deposited from github and made available on Zenodo (https://doi.org/10.5281/zenodo.5171168) for open access. The scripts in the repository outline the data analysis pipeline and skewer (v0.2.1), BMTagger (v3.101), Kraken 2 (v2.0.6), Bracken (v2.0.0), FASTP (v0.20.0), and bowtie2 (v2.4.4) were bioinformatics softwares/tools used in the analysis. RStudio (v3.6.1) was used for statistically analysis, where the phyloseq (v1.28.0), vegan (v2.5-6), and ALDEx2 (v1.16.0) packages were primarily used for analysis.

For manuscripts utilizing custom algorithms or software that are central to the research but not yet described in published literature, software must be made available to editors and reviewers. We strongly encourage code deposition in a community repository (e.g. GitHub). See the Nature Research guidelines for submitting code & software for further information.

Data

Policy information about availability of data

All manuscripts must include a data availability statement. This statement should provide the following information, where applicable:

- Accession codes, unique identifiers, or web links for publicly available datasets
- A list of figures that have associated raw data
- A description of any restrictions on data availability

All data that is not included in the manuscript or supplementary data files such as the raw FASTQ metagenomic sequencing data have been deposited in NCBI's Sequence Read Archive (SRA) under BioProject PRJNA752006 (http://www.ncbi.nlm.nih.gov/bioproject/752006) for open access. OCHA Humanitarian Data

April 2020

Exchange's datasets on DR Congo-Health Zones (https://data.humdata.org/dataset/dr-congo-health-0) and Malnutrition (https://data.humdata.org/dataset/rdc-taux-de-la-malnutrition-decembre-2019) were used to generate the map in Figure 1 and the datasets are free available to the public. The KEGG database (https://www.genome.jp/kegg/) was used as reference for identifying genes present in the dataset. All additional data used in the reported findings has been made available in the Supplementary Data Files, with references when relevant in the manuscript.

Field-specific reporting

Please select the one below that is the best fit for your research. If you are not sure, read the appropriate sections before making your selection.

- [x] Life sciences
- [ ] Behavioural & social sciences
- [ ] Ecological, evolutionary & environmental sciences

For a reference copy of the document with all sections, see nature.com/documents/nr-reporting-summary-flat.pdf

Life sciences study design

All studies must disclose on these points even when the disclosure is negative.

Sample size	The total sample size for this study involved 180 individuals from the DRC. There was not an initial power analysis to determine the sufficient sample size, however based on prior studies that have investigated differences in the gut microbiome, this sample size was appropriate for the study at hand. Each group was comprised of 30 individuals and for all comparisons each statistical was ran on 30 x 30 or larger for various comparisons used in the manuscript. The sample design was informed by notable studies at the time of sample collection, where n<30 were used/group to analyze different trends that exist of the gut microbiome of diverse populations (Schnorr et al. April 2014) (Morton et al. November 2015) (Gomez et al. March 2016).
Data exclusions	For this study, all participants that samples were taken from were included. No data was excluded during analysis.
Replication	Given that this was the first investigation into the gut flora of children in the DRC and particularly with konzo this work has yet to be exactly replicated. However, the conclusions drawn in the paper have been established using different parameters, including traditional microbial ecology measures such as the Bray-Curtis, as well as machine learning applications from different research groups in an independent fashion. Several statistical approaches were used in the study that drew the same conclusions, therefore the data was analyzed using more than one approach and led to the same overall conclusions as outlined in the methods section of the manuscript. Machine learning approaches were independently tested 10 times before final outputs were presented as described in the methods section of the manuscript. Based on the metagenomic data set, our conclusions can be replicated with the data at hand, however future work will expand on the findings presented in this study. Collectively, all attempts to replicate the presented findings in this manuscript were successful using the approaches outlined in the methods section of the manuscript.
Randomization	Participants in this study were allocated into experimental groups based on where they were collected from (geographic regions of the DRC) and whether they were affected with konzo. Given the inherent variability in the gut microbiome study participants were collected to ensure the best age matching and sex matching between groups to aid in a reduction of variability to allow conclusions to be drawn, so therefore we would not consider the selection criteria necessarily random.
Blinding	Given the nature of this study, blinding is not applicable. It was particularly important to group individuals who presented with konzo versus those individuals who were considered not affected with the disease, making blinding difficult. The same principle holds for other participants in the study, who were not affected with this particular disease, but needed to be grouped by location of sampling. During collection process controls and those affected with konzo had been previously identified and diagnosed by a team led by Dr. Desire Tshala, so the condition of the participants of this study was established prior to specimen collection. Establishing those affected from those unaffected was critical to the study design and therefore blinding was unachievable during stool collection, as those affected with konzo are phenotypically distinguishable from children who do not harbor the disease.

Reporting for specific materials, systems and methods

We require information from authors about some types of materials, experimental systems and methods used in many studies. Here, indicate whether each material, system or method listed is relevant to your study. If you are not sure if a list item applies to your research, read the appropriate section before selecting a response.

Materials & experimental systems

n/a	Involved in the study	
x		Antibodies
x		Eukaryotic cell lines
x		Palaeontology and archaeology
x		Animals and other organisms
	x	Human research participants
x		Clinical data
x		Dual use research of concern

Methods

n/a	Involved in the study	
x		ChIP-seq
x		Flow cytometry
x		MRI-based neuroimaging

Human research participants

Policy information about studies involving human research participants

Population characteristics	In total, 180 individuals were surveyed for a 7-day dietary recall questionnaire as well as provided a stool specimen for downstream microbial analysis. Study groups were collected from three different geographical regions in Western DRC including the capital of Kinshasa, Masi-Manimba and Kahemba. The breakdown of the study population of Kinshasa is as follows: Sample size = 30, Mean age = 8.7 (±1.66), Sex = 15F, 15M. The breakdown of the study population of Masi-Manimba is as follows: Sample size = 30, Mean age = 9.9 (±2.32), Sex = 15F, 15M. In Kahemba there were two focal regions in this study, regions with historically high prevalence of konzo and regions with historically low prevalence of konzo, within each region unaffected and affected individuals were selected for this study and breakdown as follows: Unaffected individuals from Kahemba low prevalence zone: Sample Size = 30, Mean age = 7.93 (± 2.32), Sex = 15F, 15M. Konzo affected individuals from Kahemba low prevalence zone: Sample size = 30, Mean age = 8.33 (± 2.67), Sex 12F, 18M. Unaffected individuals from Kahemba high prevalence zone: Sample Size = 30, Mean age = 9.03 (± 2.03), Sex = 15F, 15M. Konzo affected individuals from Kahemba high prevalence zone: Sample size = 30, Mean age = 9.63 (± 2.31), Sex = 12F, 18M.
Recruitment	This study is highly synergistic with the goals of the long-term projects and funding secured by Drs. Tshala and Boivin for investigating the molecular mechanisms associated with cassava induced neurotoxicity in the DRC. As such, individuals from the Kahemba region were recruited based on prior diagnosis of konzo by expert phenotyping of this disease. Unaffected individuals from Kahemba were not considered to have konzo at the time of sampling following the 3 main criteria for diagnosis outlined by the World Health Organization. For the Kinshasa and Masi-Manimba study population, individuals were selected from areas of convenience and who were presumed to be healthy by the local physicians who collected the samples and administered the questionnaires and consent for study participation. While we do not feel that a self-selection bias occurred, our research group did select individuals from all regions to obtain the closest age matching, to limit as much variability in downstream analysis.
Ethics oversight	This study was approved by The Ministry of Health for the Democratic Republic of Congo and the institutional review board for The Oregon Health and Sciences University.

Note that full information on the approval of the study protocol must also be provided in the manuscript.
